# Reproducibility and its confounders of CMR feature tracking myocardial strain analysis in patients with suspected myocarditis

**DOI:** 10.1007/s00330-021-08416-5

**Published:** 2021-12-21

**Authors:** Kady Fischer, Olivier L. Linder, Sophie A. Erne, Anselm W. Stark, Sarah J. Obrist, Benedikt Bernhard, Dominik P. Guensch, Adrian T. Huber, Raymond Y. Kwong, Christoph Gräni

**Affiliations:** 1 Department of Anaesthesiology and Pain Medicine, Inselspital, Bern University Hospital, University of Bern, Bern, Switzerland; 2 Department of Cardiology, Inselspital, Bern University Hospital, University of Bern, Bern, Switzerland; 3 Department of Diagnostic, Interventional and Paediatric Radiology, Inselspital, Bern University Hospital, University of Bern, Bern, Switzerland; 4 Department of Medicine, Non-Invasive Cardiovascular Imaging, Brigham and Women’s Hospital, Harvard Medical School, Cardiovascular Division, Boston, MA USA

**Keywords:** Myocarditis, Reproducibility of results, Ventricular dysfunction, Magnetic resonance imaging

## Abstract

**Objectives:**

Cardiovascular magnetic resonance feature tracking (CMR-FT) is an emerging technique for assessing myocardial strain with valuable diagnostic and prognostic potential. However, the reproducibility of biventricular CMR-FT analysis in a large cardiovascular population has not been assessed. Also, evidence of confounders impacting reader reproducibility for CMR-FT in patients is unknown and currently limits the clinical implementation of this technique.

**Methods:**

From a dual-center database of patients referred to CMR for suspected myocarditis, 125 patients were randomly selected to undergo biventricular CMR-FT analysis for 2-dimensional systolic and diastolic measures, with additional 3-dimensional analysis for the left ventricle. All image analysis was replicated by a single reader and by a second reader for intra- and inter-reader analysis (Circle Cardiovascular Imaging). Reliability was tested with intraclass correlation (ICC) tests, and the impact of imaging confounders on agreement was assessed through multivariable analysis.

**Results:**

Left and right ventricular ejection fractions were reduced in 34% and 37% of the patients, respectively. Good to excellent reliability was shown for 2D (all ICC > 0.85) and 3D (all ICC > 0.70) peak strain and early diastolic strain rate for both ventricles in longitudinal orientation as well as circumferential orientations for the left ventricle. An increased slice number improved agreement while the presence of pericardial effusion compromised diastolic strain rate agreement, and arrhythmia compromised right ventricular agreement.

**Conclusion:**

In a large clinical cohort, we could show CMR-FT yields excellent inter-reader and intra-reader reproducibility. Multi-parametric CMR-FT of the right and left ventricles appears to be a robust tool in cardiovascular patients referred to CMR.

Clinical trial registration.

ClinicalTrials.gov Identifier: NCT03470571, NCT04774549.

**Key Points**

• *Cardiovascular magnetic resonance feature tracking (CMR-FT) is an emerging technique to measure myocardial strain in cardiovascular patients referred for CMR; however, the evaluation of its reproducibility in a large cohort has not yet been performed*.

• *In a large clinical cohort, CMR-FT yields excellent inter-reader and intra-reader reproducibility for both left and right ventricular systolic and diastolic parameters*.

• *Arrhythmia and pericardial effusion compromise agreement of select FT parameters, but poor ejection fraction does not*.

**Supplementary Information:**

The online version contains supplementary material available at 10.1007/s00330-021-08416-5.

## Objectives

Myocardial strain analysis is a rapidly developing technique to investigate ventricular dysfunction. Especially in non-ischemic cardiomyopathies, such as inflammatory cardiomyopathy with heterogenous presentation, ventricular strain measurements offer a new marker for improving diagnostic accuracy and risk stratification [1–4]. Although there has been data published on the diagnostic and prognostic potential of CMR-FT [2, 3, 5], evidence on its reproducibility for analysis of different CMR-FT parameters is scarce. Specifically, reproducibility of CMR-FT has not been reported for a large cardiovascular cohort. There are some recent publications investigating reliability and agreement in control populations, and in smaller cohorts of cardiovascular patients typically with less than 30 patients [6–12]. Yet its reproducibility in a larger scale, clinical real-world patient population is still unclear especially for parameters beyond left ventricular (LV) strain 2D peak strain, including diastolic markers, measures in 3D, and the right ventricle, nor is it known which type of clinical features may impact FT analysis. During a CMR exam, there are many factors in a patient cohort present that have the potential to be confounders compromising reproducibility of the analysis that could not be assessed in a sample of healthy controls. This can include rhythmic abnormalities, pericardial effusion, ventricular abnormalities, and factors such as edema or contrast agent accumulation in fibrotic territory that may impact the signal contrast of the myocardial borders which are key for the feature tracking algorithms.

The key advantage of CMR-FT is that it is a post-processing method; thus, scan time is not extended and analysis can be applied retrospectively. The commonly published parameter is peak strain, described as the percentage of maximum deformation from diastole to systole. However, there are other markers of contractile function represented by systolic strain rate and time to peak strain. Strain rate can also be used to interpret diastolic function, by the amplitude of the early and late diastolic strain rate peaks. Therefore, CMR-FT provides a large potential for detailed assessments of ventricular function, yet for clinical implementation it is important to investigate its reproducibility in a clinical setting.

Thus, this study aimed to assess inter- and intra-reader reproducibility for biventricular CMR feature tracking in a large patient cohort of patients referred to CMR with suspected myocarditis. Secondly, it was investigated which confounders significantly compromise reader agreement in this patient setting.

## Methods

### Patient population

A total of 125 patients were randomly selected from a dual-center database of 941 patients referred for a contrast-enhanced CMR exam with the primary suspicion of myocarditis. Exclusion criteria were documented refusal of consent, cardiovascular surgery or intervention within 90 days prior to CMR, and any prior evidence or CMR characteristics for coronary artery disease, and other cardiovascular comorbidities described previously [13, 14]. The study protocol was reviewed and approved by the local Institutional Review Boards (NCT03470571) at the Brigham and Women’s Hospital, Boston, and the Inselspital, Bern University Hospital, Bern (NCT04774549).

### CMR image acquisition and analysis

**Fig. 1:**
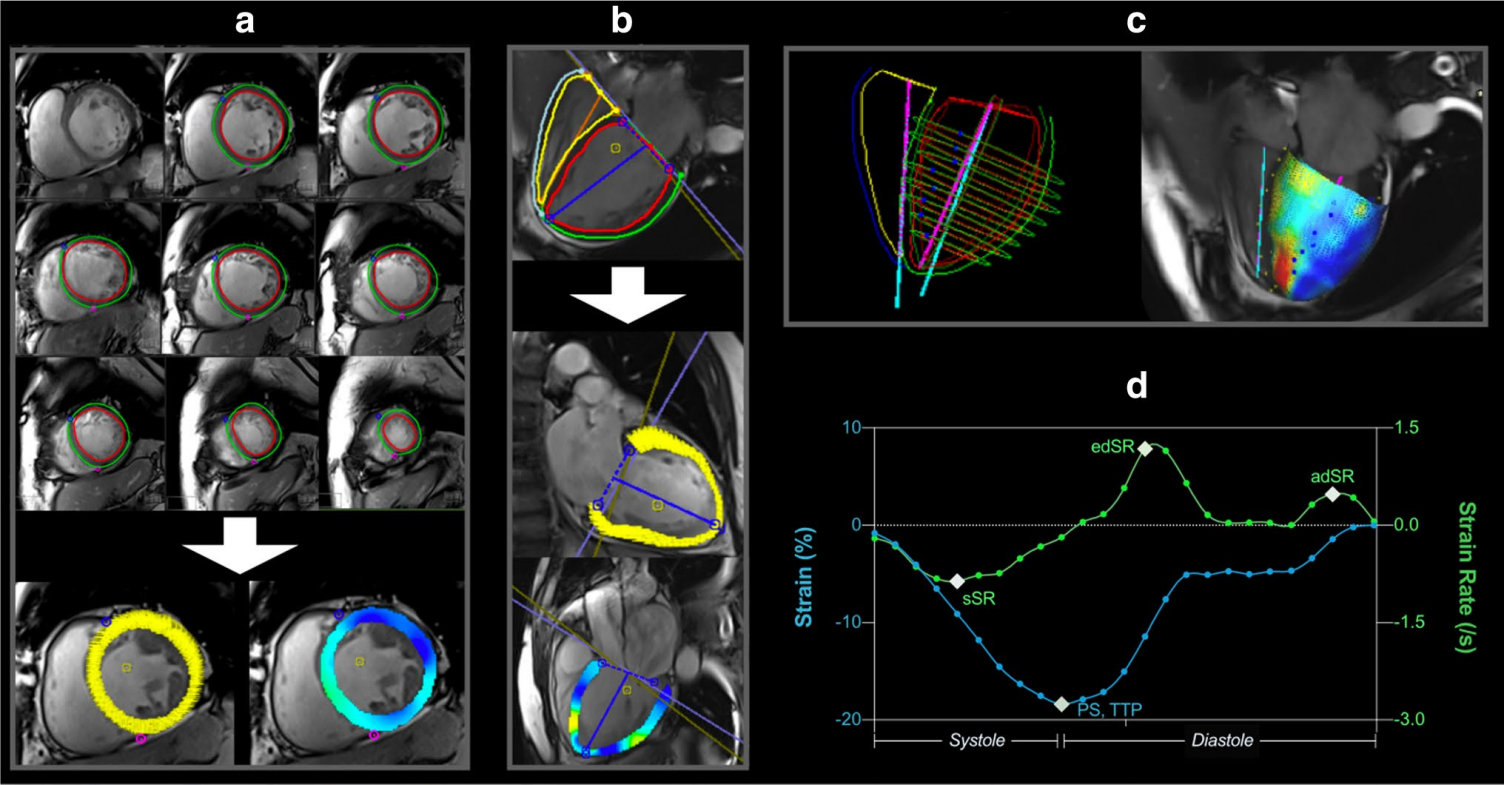
Feature tracking analysis. Feature tracking is performed on short-axis (**a**) and long-axis cinés (**b**) for 2D analysis, which can be constructed to form a 3D model (**c**). **d **A typical strain (blue) and strain rate (green) curve are shown for the longitudinal orientation, marking the key measurements; PS: peak strain, TTP: time to peak strain, sSR: systolic strain rate, edSR: early diastolic strain rate, and adSR: late diastolic strain rate

Images were obtained with a 3.0-T or a 1.5-T system (Magnetom Trio, Verio or Aera, Siemens Healthineers, GE Signa Series, GE Healthcare) [13]. Ciné images were based on clinical routine parameters used at the time of the exam, and acquisitions used in this analysis were retrospectively gated with a minimum of 25 phases (detailed in Supplemental Table 1). These cinés were acquired covering the left and right ventricle in a short-axis (SAX) stack without gap, from which biventricular circumferential and radial CMR-FT parameters were acquired along with ventricular volumes and mass. LV longitudinal strain was measured from three LV-centered long-axis (LAX) views (2-, 3-, and 4-chamber), with RV longitudinal and radial strain measured on the free-wall of the 4-chamber view only. After the placement of endocardial and epicardial contours excluding the papillaries and slices with outflow or inflow planes, deformation measurements were calculated by the feature tracking algorithms (Fig. 1, Circle Cardiovascular Imaging, version 5.9). Contours were adjusted by the reader if acquired. Two-dimensional (2D) data was acquired from the SAX and LAX planes individually, while 3D results were obtained as a result of a 3D construction by the software combining the different 2D planes. The key focus in the main text is on peak strain and early diastolic strain rate for the circumferential and longitudinal orientation. Detailed analyses for other FT parameters are provided in the supplemental information. CMR-FT datasets or individual measurement types were excluded for the following conditions: if a ciné was not acquired for the slice plane, poor angle plane, ventricular wall not fully visible, and if tracking was inadequate because of artifacts, extremely poor image quality as a result of arrhythmia, and or other tracking issues. Furthermore, the readers assessed if images were impacted by gating issues, likely caused by arrhythmia at the time of exam and for 3D models, readers categorized if the fit of 2D planes was acceptable. To assess variability of CMR-FT measurements, datasets were recoded, and the same reader re-analyzed all 125 patients blinded for intrareader assessments at least 7 days later, while a second blinded reader performed a third assessment for inter-reader analysis. CMR level III–certified readers performed for the clinical assessments of suspected myocarditis and quantified tissue characterization. Feature tracking analysis was performed by junior readers under the supervision of CMR level III analyzers.

Examinations also included the acquisition of late gadolinium enhancement (LGE) images in a short-axis stack which was quantified both by visual presence, and by the extent (% of myocardium) measured using a full-width half maximum threshold [15]. Edema was analyzed by the signal intensity ratio of the myocardium versus the major or minor pectoral skeletal muscle on T2-weighted images [13].

### Statistical methods

Mean CMR-FT measures are reported as an average of the three reads: the measurement from the primary read, the measurement from the second blinded analysis of the primary reader, along with the measurements from the second observer. Intra-reader reliability was calculated with an intra-class correlation (ICC) test using a two-way mixed model based on average measures (*k* = 2) for absolute agreement. A two-way random effects model with similar conditions was used for inter-reader reliability. ICC coefficients > 0.9 indicate excellent reliability, 0.75 to 0.9 for a good reliability, 0.5 to 0.75 for reliability, and poor reliability is represented by values < 0.5.

As the primary index of agreement, the mean absolute difference (|Δ|) of strain measures between two reads was calculated. This was performed for both inter-reader and intra-reader assessments, and also expressed as a relative difference, in comparison to the mean measurement. A repeated measures ANOVA compared 2D LV circumferential, LV longitudinal, and RV circumferential measures. A paired *t*-test compared 3D LV circumferential and longitudinal measures. Both patient- and imaging-related variables were investigated as potential confounders on reader reliability and agreement for CMR-FT analysis. To determine the impact of factors on the absolute disagreement (|Δ|) of CMR-FT analysis, univariable linear regression analysis was performed and factors and variables with *p* < 0.10 were then forwarded into a multivariable regression model to determine the strongest confounders. The forwarded variables were assessed for collinearity and removed from the multivariable model based on known relationships or by comparing models using Akaike information criterion. This was performed individually for each CMR-FT parameter presented in the manuscript.

## Results

### Population characteristics

Patient exams were performed between the years 2002–2019 (Table 1, group demographics are displayed in Supplemental Table 2). One-third of the patients had reduced left ventricular (34%) and right ventricular (37%) ejection fraction with 52% presenting with LGE. A 12-lead electrocardiogram performed prior to the CMR showed 66% had abnormal findings[14].

**Table 1:** Imaging results

	*n* = 125
Traditional CMR features	
LV ejection fraction (%)	52 ± 14
LV ejection fraction < 50%	42 (34%)
RV ejection fraction (%)	51 ± 13
RV ejection fraction < 50%	45 (37%)
Pericardial effusion	33 (26%)
Late gadolinium enhancement	65 (52%)
Extent (%)	3.9 ± 6.7
Elevated T2-ratio (> 2.0)	17/67 (25%)
Imaging features	
GE scanner	51 (41%)
Siemens scanner	74 (59%)
1.5 Tesla	68 (54%)
3 Tesla	57 (46%)
≥ 30 phases	48 (38%)
Number of SAX slices per patient	8 [7-9]
Number of LAX slices per patient	3 [2-3]
Poor gating	16 (13%)
Time between ciné series (min)	23 [9-33]
Ciné images acquired:	
• Pre-contrast • Mixed pre- and post-contrast • Post-contrast	31 (25%) 27 (22%) 67 (54%)

Data are mean ± SD, median [interquartile range] or frequency, *n*, and percentage (%)

### Inclusion of CMR-FT data

2D longitudinal measurements were acquired in the highest proportion of patients, with up to 124/125 patients analyzed (99%). Analysis of SAX slices was performed in 122 (98%) of patients. LV-3D measurements were performed in 120 (96%) patients, while inclusion was lowest for RV analysis. A maximum of 106 (85%) patients were analyzed for RV FT (reasons for exclusion detailed in Supplemental Fig. 1).

## Reader Reliability and Agreement

**Fig. 2:**
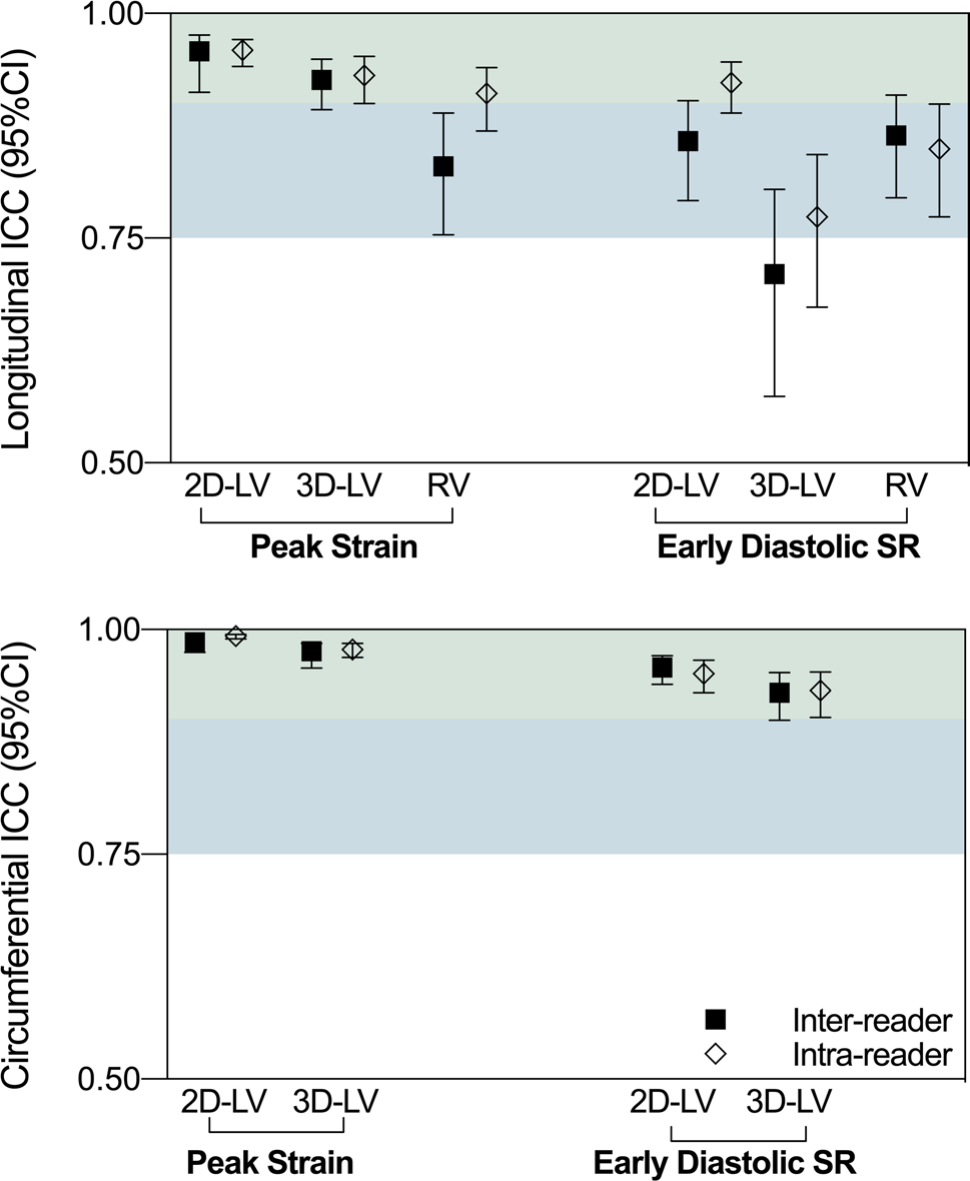
Intraclass correlation coefficients. Inter-reader (square) and intra-reader (diamond) intraclass correlation coefficients (ICC) and 95% confidence intervals demonstrated significant reliability (all *p* < 0.05). Green zone: excellent reliability (≥ 0.90), blue zone: good reliability (0.75–0.90). LV: left ventricle, RV: right ventricle

Good to excellent inter-reader and intra-reader ICC coefficients were observed for all systolic parameters and early diastolic strain rates (Supplemental Tables 3–5). As shown in Fig. 2, LV peak strain for both 2D and 3D was excellent with slightly better ICC observed for the circumferential orientation (GCS) over longitudinal (GLS). Moreover, the relative disagreement for 2D GCS was 6% and 4% for inter-reader and intra-reader respectively, both, which were significantly less than the 2D GLS relative disagreement of 10% and 9% (*p* < 0.001 for both inter- and intra-reader, Table 2). The same observation was statistically significant with 3D measurements (*p* < 0.001). For the RV, both inter- and intra-reader ICCs were good as well, although RV GLS showed higher relative disagreement than LV GCS (*p* < 0.001) and LV GLS (*p* = 0.025 for inter-reader, and non-significant *p* = 0.068 for intra-reader).

**Table 2:** Reader reproducibility

		Inter-reader			Intra-reader		
	Mean + SD	ICC (95%CI)	Absolute Agreement *|*Δ*|*	Relative Agreement %	ICC (95%CI)	Absolute Agreement *|*Δ*|*	Relative Agreement **%**
Global peak strain (%)							
2D-LV longitudinal	− 13.2 ± 4.0	0.96 (0.91–0.98)*	1.3 ± 1.1	10 ± 9	0.96 (0.94–0.97)*	1.1 ± 1.2	9 ± 8
3D-LV longitudinal	− 10.9 ± 4.0	0.93 (0.89–0.95)*	1.6 ± 1.5	17 ± 26	0.93 (0.90–0.96)*	1.5 ± 1.4	18 ± 27
RV longitudinal	− 18.5 ± 5.4	0.83 (0.75–0.90)*	2.7 ± 3.0	16 ± 19	0.91 (0.87–0.94)*	2.1 ± 2.4	13 ± 17
2D-LV circumferential	− 14.7 ± 4.7	0.99 (0.98–0.99)*	0.8 ± 0.7	6 ± 6	0.99 (0.99–0.99)*	0.6 ± 0.6	4 ± 5
3D-LV circumferential	− 16.2 ± 5.1	0.98 (0.96–0.99)*	1.2 ± 1.1	8 ± 12	0.98 (0.97–0.99)*	1.1 ± 1.1	7 ± 8
Early diastolic strain rate (/s)							
2D-LV longitudinal	0.71 ± 0.25	0.86 (0.79–0.90)*	0.13 ± 0.13	20 ± 22	0.92 (0.89–0.95)*	0.11 ± 0.10	16 ± 16
3D-LV longitudinal	0.65 ± 0.26	0.71 (0.57–0.80)*	0.16 ± 0.20	24 ± 30	0.77 (0.67–0.84)*	0.17 ± 0.19	26 ± 25
RV longitudinal	1.40 ± 0.90	0.86 (0.80–0.91)*	0.54 ± 0.87	25 ± 27	0.85 (0.77–0.90)*	0.50 ± 0.73	21 ± 19
2D-LV circumferential	0.84 ± 0.33	0.94 (0.91–0.96)*	0.08 ± 0.14	10 ± 16	0.95 (0.93–0.97)*	0.08 ± 0.13	10 ± 16
3D-LV circumferential	0.97 ± 0.38	0.93 (0.90–0.95)*	0.12 ± 0.16	14 ± 21	0.93 (0.90–0.95)*	0.13 ± 0.16	15 ± 21

The measurement for each parameter is shown as mean ± SD averaged from all three reads along with the intraclass correlation coefficient (ICC) along with the 95% confidence intervals (CI), *p < 0.001. The disagreement between reads is reported as the mean ± SD absolute difference (|Δ|), and as relative disagreement calculated as the percentage of the |Δ| against the mean measurement. *LV* left ventricle, *RV* right ventricle

**Fig. 3:**
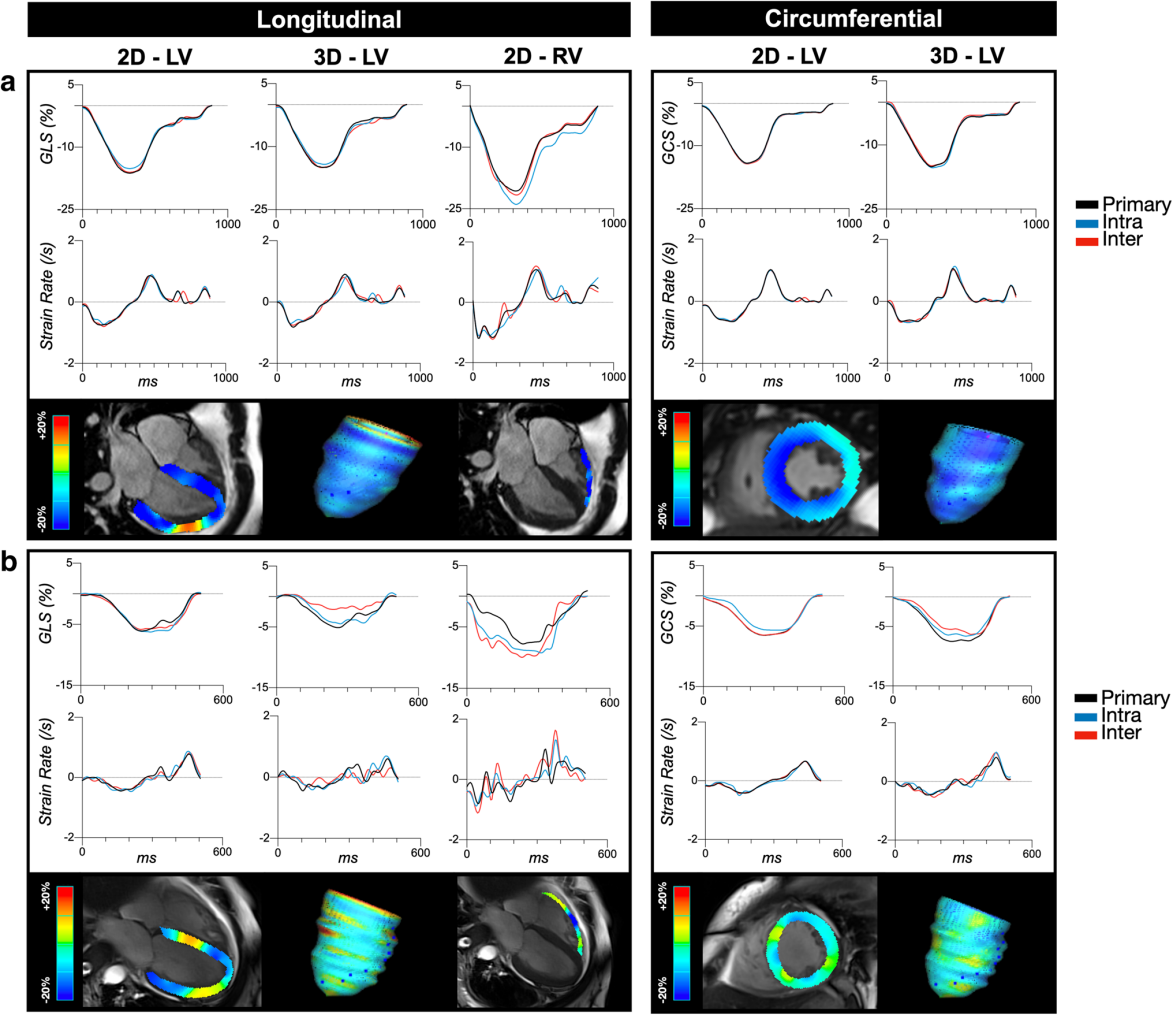
Examples of agreement. **a **Strain and strain rate curves are similar between all three reads and demonstrating excellent agreement in this 30-year-old patient with a resting heart rate of 65 bpm and a late gadolinium extent of 27%. **b **The second case shows poorer agreement, especially in the right ventricle (RV) in comparison to the left (LV) from a 41-year-old patient with a resting heart rate of 109 bpm who had 5% late gadolinium enhancement

For diastolic measures, 2D and 3D LV early diastolic strain rate reliability in circumferential orientation was excellent with a relative disagreement ranging from 10.3 to 15.2% of the mean early diastolic strain rate. Similar to peak strain, diastolic markers were best in the circumferential orientation for both 2D and 3D measures of the LV (*p* < 0.001, Fig. 3). In the longitudinal orientation, a good ICC was observed for 2D LV and RV measures resulting in a relative disagreement of 16–26%. However, as visualized in Fig. 2, ICC was poorest for 3D longitudinal early diastolic strain rate.

### Factors impacting agreement

Multivariable analysis showed that for the majority of 2D and 3D LV peak strain and diastolic measures, an increase in slices independently improved agreement (Tables 3 and 4). Agreement was better for ventricles with higher end-diastolic volumes and reduced ventricular function shown by attenuated feature tracking measures and ejection fractions. For LV 3D early diastolic strain rate, the presence of pericardial effusion compromised agreement both circumferentially and longitudinally. Sixteen (13%) of the images were deemed by the readers to be impacted by poor gating, likely due to arrhythmia, and this was the only significant confounder for the agreement of RV GLS in the multivariable model. For the RV diastolic parameters, independent confounding factors differed between inter-reader and intra-reader models, with agreement better at 1.5 T versus 3 T, at lower heart rates and at reduced early diastolic strain rates.

**Table 3:** Key confounders impacting reader agreement for longitudinal feature tracking

	Inter-reader				Intra-reader			
	Univariable		Multivariable		Univariable		Multivariable	
	β	*p*	β	*p*	β	*p*	β	*p*
Peak strain								
Δ2D LV								
Baseline 2D-GLS	− 0.71	0.027			− 1.09	< 0.001	− 0.09	< 0.001
LV ejection fraction	3.01	0.009	0.01	0.047	3.20	0.003		
End diastolic volume	− 12.4	0.019			− 18.1	< 0.001		
Slice number	− 0.10	0.037			− 0.13	0.004	− 0.43	0.028
LGE (%)	− 1.17	0.033						
Phases (≥ 30)					− 0.47	0.044		
Temporal resolution*					1.25	0.073		
Δ3D LV								
Siemens (vs. GE)	− 0.06	0.035			− 0.06	0.091		
Heartrate	− 2.87	0.004						
Slice number	− 0.06	0.058	− 0.49	0.035	− 0.11	0.008	− 0.46	0.030
LGE (%)	− 0.68	0.086			− 0.87	0.057		
End diastolic volume	− 9.42	0.014			− 8.65	0.052		
LV ejection fraction	2.19	0.010			2.14	0.032		
Phases (≥ 30)	0.065	0.026			0.08	0.026		
Temporal resolution*	1.78	< 0.001						
Year					− 0.60	0.030		
Poor 3D reconstruction	0.02	0.063						
ΔRV								
3 Tesla	0.05	0.048						
Gating (arrhythmia)	0.02	0.037	2.12	0.030	0.03	0.010	2.02	0.008
Temporal resolution*					0.781	0.040	0.05	0.089
RV stroke volume					1.98	0.094		
Early diastolic strain rate								
Δ2D LV								
Heartrate	26.0	0.033			30.9	0.043	0.001	0.015
Temporal resolution*	− 12.9	0.048			− 14.3	0.082		
Slice number	− 0.95	0.012	− 0.02	0.015	− 1.12	0.033	− 0.04	0.019
Stroke volume	− 39.1	0.043	− 0.002	0.010				
LGE extent (%)	− 9.76	0.048	− 0.003	0.073	− 13.2	0.029	− 0.003	0.057
LGE presence	− 0.66	0.066						
Baseline 2D-edSR	0.296	0.091			0.62	0.005		
Siemens (vs GE)					− 0.88	0.045		
Post contrast agent					0.66	0.081	0.55	0.012
Phases (≥ 30)					0.84	0.056		
Δ3D LV								
Poor 3D construction	0.20	0.014	0.32	0.005				
Pericardial effusion	0.40	0.066	0.08	0.042	0.61	0.005	0.11	0.002
LGE presence	− 0.49	0.055	–	–	–	–		
Baseline 3D-edSR	0.36	0.003	0.25	0.002	0.57	< 0.001	0.31	< 0.001
ΔRV								
3-Tesla	1.19	0.002	0.10	0.016				
Siemens (vs GE)	0.46	0.083						
Post contrast agent	0.461	0.051						
Phases (≥ 30)	− 0.52	0.053						
Pericardial effusion	0.48	0.048						
Heartrate					23.9	0.005	0.003	0.024
Temporal resolution*					–9.16	0.044		
Baseline RV-edSR					0.47	0.005	0.14	0.021

Variables that demonstrated a potential impact (*p* < 0.10) on agreement (|Δ|) are displayed

*edSR* early diastolic strain rate, *GLS* global longitudinal peak strain, *LGE* late gadolinium enhancement, *LV* left ventricle, *RV* right ventricle

^*^Individual temporal resolutions for the retrospectively gated ciné’s were calculated as the RR-interval/cardiac phases

**Table 4:** Key confounders impacting reader agreement for circumferential feature tracking

Factor	Inter-reader				Intra-reader			
	Univariable		Multivariable		Univariable		Multivariable	
	β	*p*	β	*p*	β	*p*	β	*p*
Peak strain								
Δ2D LV								
*LV ejection fraction*	4.47	0.010	0.01	0.014				
*Stroke volume*	5.53	0.090						
*Myocardial mass*	11.9	0.017	0.007	< 0.001				
*LGE presence*	–0.14	0.020	0.35	0.009				
*Gating (arrhythmia)*	0.09	0.034	0.43	0.023				
*Slice number*	–0.46	0.008	–0.12	0.005	–0.56	0.012	–0.07	0.048
*Year*					–1.38	0.028		
Siemens (vs GE)					–0.14	0.078		
*Phases (*≥ *30)*					00.17	0.026		
Δ3D LV								
*Slice number*	–0.38	0.001	–0.15	0.064	–0.29	0.015	–0.17	0.016
*End diastolic volume*	–11.4	0.031						
*LV ejection fraction*	1.93	0.100						
*Poor 3D-reconstruction*	0.03	0.028	0.99	0.085				
Temporal resolution*	1.34	0.077	0.02	0.052				
Early diastolic strain rate								
Δ2D LV								
*Heart rate*					20.9	0.080	0.001	0.080
*LGE presence*	–0.58	0.075	0.05	0.075				
Δ3D LV								
*Year*	7.0	0.002						
Siemens (vs GE)	0.49	0.086						
*Post contrast agent*	–0.43	0.068						
*Phases (*≥ *30)*	–0.52	0.069						
*Time between images*					–14.8	0.068		
*Pericardial effusion*					0.59	0.020	0.11	0.048

Variables that demonstrated a potential impact (*p* < 0.10) on agreement (|Δ|) are displayed

*LGE* late gadolinium enhancement, *LV* left ventricle, *RV* right ventricle

^*^Individual temporal resolutions for the retrospectively gated ciné’s were calculated as the RR-interval/cardiac phases

## Discussion

The present study shows that biventricular strain analysis using CMR-FT is highly reproducible for both systolic and diastolic function in a cardiovascular patient population referred for clinically indicated CMR for suspected myocarditis. In the multivariable analysis, factors present during a clinical exam are independently likely to compromise reader agreement for the individual parameters. Compared to healthy volunteer studies, which are not afflicted by clinical factors that can impact image acquisitions such as arrhythmia, pericardial effusion, or myocardial injury[13], we could show in a large cohort and a real-world clinical setting, that left- and right-heart peak strain and strain rates are consistently detected by between measurements with excellent agreement. Out of the different parameters, peak systolic strain yielded the highest reliability while other factors including time to peak strain and strain rate values are slightly more compromised. We observed that edema did not significantly impact tracking or reader agreement in our patient population with suspected myocarditis. Interestingly, the presence of LGE and consequently lower ventricular function was associated with better agreement. This is likely due to the fact that these patients with ventricular dysfunction have less rapid myocardial movement (lower tissue velocities) thus allowing the tracking algorithms to follow the myocardial features more accurately. On the other hand, the presence of pericardial effusion and arrhythmia did compromise agreement, especially for diastolic function and right ventricular analysis respectively. As image quality of ciné CMR has evolved over the past decade, another key factor we considered was the year images were acquired. Despite the fact that both pulse sequence design and signal-to-noise ratio of ciné imaging have improved in recent years, chronological age (years) of the exam did not have a significant impact on the FT agreement. This is especially relevant to the multiple studies that had applied CMR-FT retrospectively and investigate long-term outcomes [2, 3]. While focus is on peak strain, with some papers introducing diastolic strain rates, there is room to investigate the multiple markers acquired simultaneously from this analysis. This includes displacement and velocity measurements, along with time to peak strain to assess post-systolic shortening and mechanical dispersion [16, 17]. Thus, we have provided reliability analyses for these markers as well to support future utilization of these measures.

### Comparisons of orientations

For both 2D and 3D systolic and diastolic measures, circumferential measures showed the highest reliability and agreement in comparison to longitudinal [6, 18]. An explanation for a higher reproducibility with GCS in CMR studies might be related to our observation of improved agreement in SAX stacks with numerous slices. A full SAX stack acquired in CMR will often incorporate 3–4 fold more slices than a typical 3-planar LAX acquisition. Thus, in a clinical setting where arrhythmia, poor gating, and rapid heart rate may be present, the utilization of more slices can allow any errors to be averaged out better whereas the fewer slices used for LAX analysis may be more exposed to these issues. Consequently, it would be advisable to perform analysis on the maximum data as possible and artificial intelligence techniques now allow this to be performed without a significant increase in workload. It is important to note in our study the reliability for longitudinal markers was still excellent, and GLS in particular is still often most commonly used for diagnostic and prognostic reasons [3, 5]. As circumferential and longitudinal fibers compose different regions of the myocardium, assessment of these orientations may be used in the future to investigate different disease processes.

### Left ventricular diastolic function

Impaired diastolic function can lead to the onset of symptoms and cardiovascular events [19], especially in cohorts where systolic dysfunction is not overtly impaired. We also recently showed that diastolic strain rate was a significant prognostic marker as well, especially in patients with an LVEF > 40% [3]. While awareness for the clinical significance of this marker is rising, little is known about its reproducibility with CMR-FT. In 20 patients with a myocardial infarction, Nazir et al. showed 2D early diastolic strain rate measurements were reproducible by CMR-FT at both 1.5 and 3 T, comparable to MRI tagging [7]. Here we observed for 2D and 3D left ventricular measurements, mostly good to excellent agreement for early diastolic strain rate. When looking at patient factors, 2D agreement was improved in patients with LGE enhancement and 3D agreement was improved in patients with poor diastolic dysfunction. However, in the presence of pericardial effusion or a poor alignment of the 3D construction, diastolic strain rates were less reproducible.

### 2D versus 3D reproducibility

With advancing technology, 3D imaging is available with the goal of providing better coverage of the heart. In an independent acute myocarditis cohort, Gatti et al. reported that peak strain and systolic and diastolic strain rates were impaired in acute myocarditis for both 2D and 3D CMR-FT analysis [20]. We also observed a moderate to good ICC for many 3D parameters in our patient cohort; however, these were slightly poorer in comparison to the 2D measurements with a larger relative disagreement. This finding is contradictory to previous findings by Liu et al. in 100 healthy controls, where 3D FT-CMR yielded more reproducible analysis [9]. Unlike true 3D block acquisitions that are acquired within a single measurement, the 3D models used for CMR-FT analysis are only constructions of multiple 2D short-axis and 2D long-axis planes. Thus, the 3D construction is dependent on accurate fitting of multiple images acquired in different acquisitions. Ciné acquisitions were spread out across the exam, extending to 59 min in some cases. Especially for the 3D analysis, this is not ideal as it is likely there is minor patient movement over the course of an hour. Furthermore, patients are more likely to have issues with maintaining breath-holds in the same end-expiratory position, and arrhythmia and poor gating can impact reconstruction as well. Consequently, we observed that a mismatched fit did compromise 3D GCS and GLS inter-reader agreement. 3D assessments would likely be improved if SAX and LAX acquisitions are acquired in sequence.

### Right ventricular feature tracking

RV strain is increasingly incorporated as a marker of RV diagnostics [21, 22]. Generally, studies with samples of under 20 controls or patients investigating RV reproducibility have shown fair results for free wall peak strain and diastolic strain rate [8, 22–24]. With a sample of 125 patients, we observed good reliability for RV peak strain and diastolic strain rate, demonstrating both systolic and diastolic functions of the RV can be reliably measured in a patient population. However, we excluded a higher proportion of RV FT measurements compared to the LV. During imaging exams, the LV may be prioritized over the RV and we observed minor wrap or artifact in the RV that may not significantly hamper visual assessments of function but did create tracking errors. Similarly, multiple images were excluded because of pulsatile flow artifacts over the RV, especially at the base of the RV free wall. Tricuspid valve excursion is higher than the mitral valve, resulting in rapid regional movement in the RV. In combination with these pulsatile artifacts, tracking was impacted. Moreover, only RV agreement was compromised by the 3 T magnetic strength in comparison to 1.5 T, and it is known pulsatile flow artifacts can be worse at the higher field because of more susceptibility. The other factor that impacted RV analysis was arrhythmia and poor gating. As RV analysis was restricted to shortening in the LAX and thus only conducted in one plane, if this image has poor image quality or tracking, there are not other slices or planes to compensate as observed in the LV.

## Limitations

Our study inherits several limitations. We investigate reader-reader reproducibility of one single vendor-specific CMR-FT application; thus, our results may not be translated to other software. As feature tracking techniques are advancing, updated software versions are continuously released. For this analysis, we used a version that relies on contouring of diastolic contours only. The addition of guiding contours or corrections in other phases where tracking is not ideal would likely improve measurements and reliability. Finally, effort is still needed to achieve a technical and clinical standardization.

## Conclusion

CMR-FT yields excellent inter-reader and intra-reader reader reliability and agreement for biventricular peak systolic strain and early diastolic strain rates in a patient group with suspected myocarditis. Clinical factors, i.e., pericardial effusion and arrhythmia, affect reader agreement during the CMR acquisition. Therefore, these clinical factors should be taken into account when readers interpret CMR-FT results. CMR-FT appears to be a highly reproducible method in a cohort of cardiovascular patients referred for clinically indicated CMR.

## Supplementary Information

Below is the link to the electronic supplementary material.

Supplementary file1 (PDF 2979 kb)

## Abbreviations

CMR-FT: Cardiovascular magnetic resonance feature tracking
edSR: Early diastolic strain rate
GCS: Global circumferential strain
GLS: Global longitudinal strain
GRS: Global radial strain
ICC: Intraclass correlation coefficient
LAX: Long-axis
LGE: Late gadolinium enhancement
LVEF: Left ventricular ejection fraction
RVEF: Right ventricular ejection fraction
SAX: Short-axis

## Declarations

### Guarantor

The scientific guarantor of this publication is Christoph Gräni (Christoph.graeni@insel.ch).

### Statistics and biometry

No complex statistical methods were necessary for this paper.

### Study subjects or cohorts overlap

Portions of this cohort from one of the sites (Department of Medicine, Brigham and Women’s Hospital, Harvard Medical School, Boston, MA, USA) have been previously published for the aims of prognostication of imaging and clinical markers. The current manuscript now includes patients from a larger combined registry which is now dual-center. Additionally, there is no overlap with the aims of this submission and previous publications. (https://doi.org/10.1016/j.jacc.2017.08.050, 10.1007/s10554.019.01552.6, 10.1186/s12968.019.0520.0, 10.1016/j.jcmg.2020.04.025).

### Methodology

• Retrospective

• Multi-center

• Observational study
