# Supplementary material for: Reproducibility and its confounders of CMR feature tracking myocardial strain analysis in patients with suspected myocarditis
Source: Eur Radiol. 2021 Dec 21;32(5):3436–46. doi: 10.1007/s00330-021-08416-5 (PMC9038796; doi:10.1007/s00330-021-08416-5)
Supplement: Supplementary file 1 — Supplementary file1 (PDF 2979 kb) [file 330_2021_8416_MOESM1_ESM.pdf]

SUPPLEMENTAL MATERIAL

Reproducibility and its Confounders of CMR Feature Tracking Myocardial Strain Analysis in Patients with Suspected Myocarditis

TABLE OF CONTENTS

Supplemental Table 1: Sequence Parameters 2

Supplemental Figure 1: Data Inclusion 3

Supplemental Figure 2: Heat Map of Agreement Confounders for 2D LV Parameters 4

Supplemental Table 2: Additional Patient Demographics 5

Supplemental Table 3: 2D Left Ventricular Agreement 6

Supplemental Table 4: 3D Left Ventricular Agreement 7

Supplemental Table 5: Right Ventricular Agreement 8

Supplemental Methods

Sample size rationale

The sample size of n=125 was determined based on the rationale that up to 20% of data may be excluded, especially for right ventricular, 3D and diastolic measures, and a final data of 100 subjects is in line with the sample size used for reproducibility assessments of CMR feature tracking in healthy controls by Liu et al[9], while allowing for multivariable analysis.

Image acquisition

All ciné's were acquired with standard clinical ECG gated (retrospective) balanced steady state free precession sequences, based on the parameters displayed in Supplemental Table 1. Due to the retrospective nature of the sequence, temporal resolution was defined by the pre-specified phase number per cardiac cycle and the individuals RR-interval (ie. heart rate). Short-axis slices were acquired for the entire ventricles without a gap (distance factor: 0%), and long-axis views were acquired in two-, three- and four-chamber views.

Supplemental Table 1: Sequence Parameters

	**1.5T -GE**	**1.5T - Siemens**	**3T - Siemens**
**Sequence Name**	FIESTA	TrueFISP	TrueFISP
**Matrix**	192*160	208*126	192*144
**Bandwidth (Hz/Px)**	977	925	930
**Slice thickness (mm)**	8.0	8.0	8.0
**Flip Angle (°)**	45	50	45
**Echo Time, TE (ms)**	1.50	1.10	1.31
**Repetition Time, TR (ms)**	54 (3.4)	42 (2.6)	54 (3.6)
**Phase Number**	30	25-30	25
**Calculated temporal resolution** (for an RR-interval of 1000ms)	33ms	33-40ms	40ms

Detailed strain analysis

Analysis was performed on the short axis stack and three long axis views, as displayed in Figure 1. Readers visually determined the end-diastolic phase. Using an automatic contouring tool, endocardial and epicardial contours were placed on the diastolic phase and reviewed by the readers and corrected if required. The contours excluding papillaries and trebicles, as well as outflow and inflow planes. Afterwards the feature tracking analysis was run using the end-diastolic frame as reference using the software from Circle Cardiovascular Imaging, version 5.9. Readers then visually verified the tracking, and if required readers adjusted the contours and repeated the above steps. If tracking was not acceptable after adjustments, the data set was excluded as shown in Supplemental Figure 1.

Supplemental Figure 1: Data Inclusion

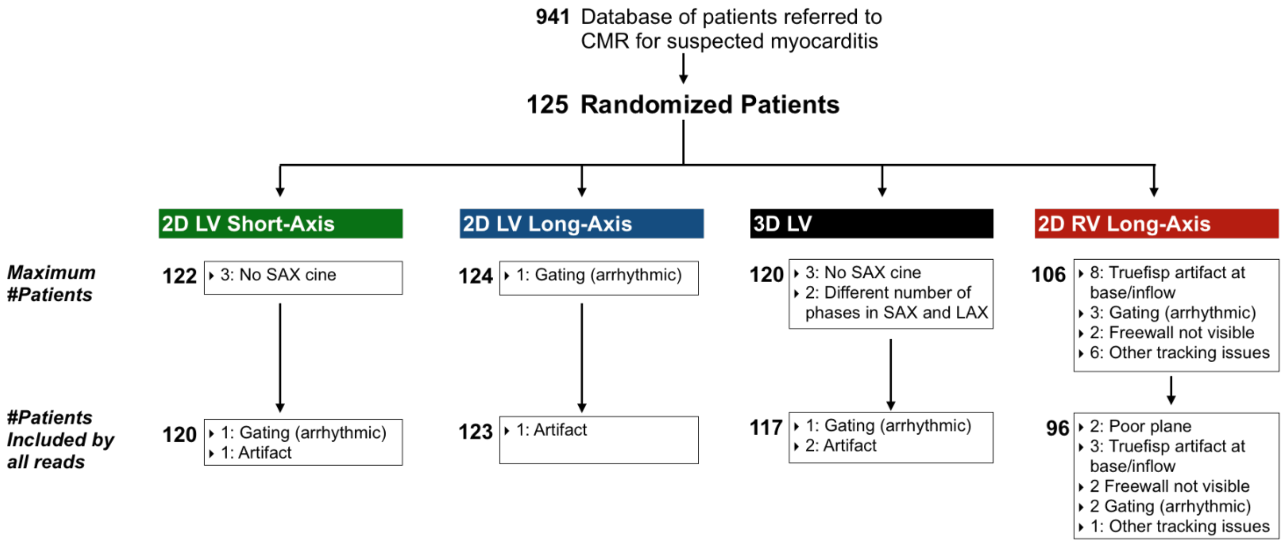

Inclusion per plane was first displayed by the maximum number of patients available (those who all three reads agreed to exclude), then by patients in which there was disagreement in exclusion, resulting in the final number of patients all reads agreed to include. 2D: two-dimension, 3D: three-dimension, LAX: long-axis, LV: Left ventricle, RV: Right Ventricle, SAX: short-axis.

Supplemental Figure 2: Heat Map of Agreement Confounders for 2D LV Parameters

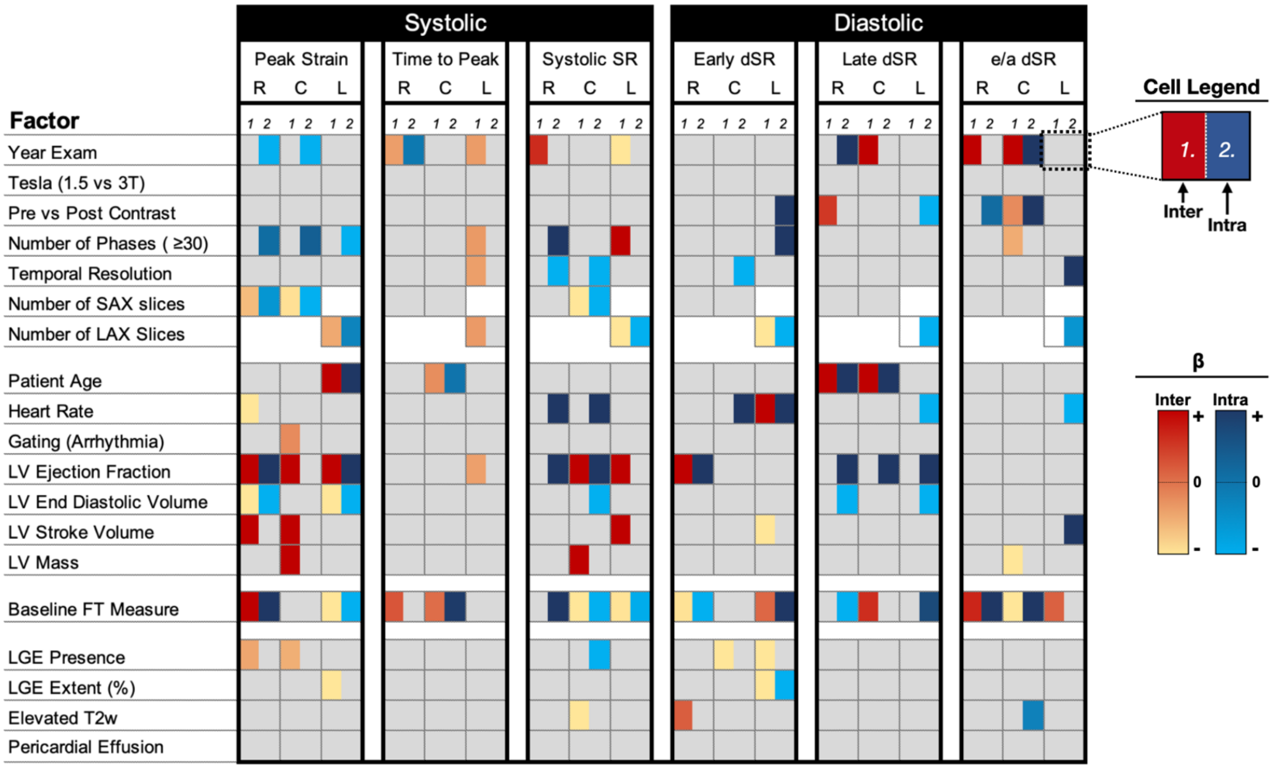

The heat map depicts beta estimates from significant univariate regression between potential confounders and the difference in agreement (|Δ|) for 2D left ventricular feature tracking measurements for each orientation, R: radial, C: Circumferential. For each measurement, the left half-cell represents the assessment of inter-reader agreement and the right represents intra-reader agreement. Non-significant assessments are marked by a grey square.

Supplemental Table 2: Additional Patient Demographics

Age (years)	45 ± 16
Sex (females)	51 (41%)
Body mass index (kg/m^2)	27.5 ± 6.4
**Medical History**	
Diabetes mellitus	8 (6%)
Hypertension	27 (22%)
Heart failure	19 (15%)
Cholesterolemia	21 (17%)
Current smoker	19 (15%)
New York Heart Association	
Grade II	15 (12%)
Grade III	16 (13%)
Grade IV	3 (2%)
**Medications**	
Beta blockers	38 (30%)
Calcium Channel Antagonists	6 (5%)
ACE Inhibitors	38 (30%)
Statins	20 (16%)
Acetylsalicylic acid	32 (26%)
Diuretics	21 (17%)

Mean±SD or frequency (percentage) are displayed for patient characteristics at the time of the CMR exam.

Supplemental Table 3: 2D Left Ventricular Agreement

Factor	Average Measure	Inter-reader ICC (95% CI)	Inter-reader p-value	Inter-reader Absolute Disagreement |Δ|	Intra-reader ICC (95% CI)	Intra-reader p-value	Intra-reader Absolute Disagreement |Δ|
**Peak Strain (%)**							
Radial (SAX)	23.5 ± 9.9	0.99 (0.97-0.99)	<0.001	1.8 ± 1.6	0.99 (0.99-0.99)	<0.001	1.2 ± 1.3
Circumferential	-14.7 ± 4.7	0.99 (0.98-0.99)	<0.001	0.8 ± 0.7	0.99 (0.99-0.99)	<0.001	0.6 ± 0.6
Longitudinal	-13.3 ± 3.9	0.96 (0.91-0.98)	<0.001	1.3 ± 1.1	0.96 (0.95-0.97)	<0.001	1.1 ± 1.2
**Time to Peak Strain (ms)**							
Radial (SAX)	304± 53	0.96 (0.94-0.97)	<0.001	9 ± 19	0.98 (0.97-0.98)	<0.001	7 ± 14
Circumferential	307 ± 55	0.88 (0.83-0.92)	<0.001	14 ± 36	0.91 (0.88-0.94)	<0.001	11 ± 32
Longitudinal	323 ± 54	0.71 (0.59-0.80)	<0.001	32 ± 49	0.83 (0.75-0.88)	<0.001	27 ± 40
**Systolic Strain Rate (/s)**							
Radial (SAX)	1.32 ± 0.58	0.99 (0.98-0.99)	<0.001	0.11 ± 0.09	0.99 (0.98-0.99)	<0.001	0.09 ± 0.09
Circumferential	-0.85 ± 0.28	0.98 (0.97-0.99)	<0.001	0.06 ± 0.05	0.97 (0.96-0.98)	<0.001	0.06 ± 0.07
Longitudinal	-0.76 ± 0.20	0.88 (0.79-0.92)	<0.001	0.11 ± 0.11	0.88 (0.83-0.92)	<0.001	0.11 ± 0.11
**Early Diastolic Strain Rate (/s)**							
Radial (SAX)	-1.41 ± 0.70	0.96 (0.94-0.97)	<0.001	0.17 ± 0.23	0.97 (0.96-0.98)	<0.001	0.14 ± 0.20
Circumferential	0.84 ± 0.33	0.94 (0.91-0.96)	<0.001	0.08 ± 0.14	0.95 (0.93-0.97)	<0.001	0.08 ± 0.13
Longitudinal	0.71 ± 0.25	0.86 (0.79-0.90)	<0.001	0.13 ± 0.13	0.92 (0.89-0.95)	<0.001	0.11 ± 0.10
**Late Diastolic Strain Rate (/s)**							
Radial (SAX)	-0.48 ± 0.29	0.95 (0.92-0.96)	<0.001	0.06 ± 0.12	0.97 (0.96-0.98)	<0.001	0.04 ± 0.08
Circumferential	0.43 ± 0.23	0.93 (0.90-0.95)	<0.001	0.05 ± 0.10	0.96 (0.93-0.97)	<0.001	0.04 ± 0.08
Longitudinal	0.49 ± 0.17	0.76 (0.68-0.86)	<0.001	0.11 ± 0.11	0.80 (0.71-0.87)	<0.001	0.11 ± 0.10
**Diastolic Strain Rate ratio**							
Radial (SAX)	3.85 ± 2.35	0.92 (0.88-0.95)	<0.001	0.62 ± 1.00	0.92 (0.88-0.95)	<0.001	0.56 ± 1.04
Circumferential	2.61 ± 1.71	0.89 (0.84-0.93)	<0.001	0.41± 0.80	0.90 (0.85-0.93)	<0.001	0.38 ± 0.77
Longitudinal	1.62 ± 0.76	0.77 (0.66-0.85)	<0.001	0.46 ± 0.45	0.75 (0.63-0.84)	<0.001	0.45 ± 0.46
**Displacement (mm)**							
Radial (SAX)	4.50 ± 1.37	0.98 (0.97-0.99)	<0.001	0.25 ± 0.28	0.99 (0.98-0.99)	<0.001	0.19 ± 0.22
Longitudinal	3.01 ± 1.12	0.93 (0.90-0.95)	<0.001	0.44 ± 0.39	0.95 (0.92-0.96)	<0.001	0.42 ± 0.34
**Time to Displacement (ms)**							
Radial (SAX)	309 ± 58	0.97 (0.95-0.98)	<0.001	9 ± 19	0.97 (0.96-0.98)	<0.001	8 ± 17
Longitudinal	307 ± 68	0.76 (0.66-0.84)	<0.001	40 ± 58	0.79 (0.71-0.86)	<0.001	40 ± 53
**Systolic Velocity (m/s)**							
Radial (SAX)	26.0 ± 7.7	0.98 (0.97-0.99)	<0.001	1.6 ± 1.7	0.98 (0.97-0.98)	<0.001	1.7 ± 1.7
Longitudinal	19.9 ± 6.4	0.80 (0.72-0.86)	<0.001	4.4 ± 4.1	0.83 (0.76-0.88)	<0.001	4.0 ± 3.6
**Early Diastolic Velocity (m/s)**							
Radial (SAX)	-26.7 ± 10.5	0.98 (0.97-0.99)	<0.001	1.9 ± 2.0	0.98 (0.98-0.99)	<0.001	1.8 ± 2.1
Longitudinal	-19.1 ± 8.6	0.88 (0.82-0.92)	<0.001	4.4 ± 4.4	0.91 (0.87-0.93)	<0.001	4.1 ± 3.7
**Late Diastolic Velocity (m/s)**							
Radial (SAX)	-12.0 ± 5.7	0.92 (0.86-0.95)	<0.001	1.5 ± 2.6	0.95 (0.92-0.97)	<0.001	1.2 ± 2.2
Longitudinal	-12.8 ± 5.0	0.70 (0.55-0.80)	<0.001	3.6 ± 3.9	0.62 (0.43-0.75)	<0.001	3.9 ± 4.3
**Diastolic Velocity ratio**							
Radial (SAX)	2.92 ± 2.03	0.90 (0.85-0.93)	<0.001	0.46 ± 1.01	0.93 (0.90-0.95)	<0.001	0.36 ± 0.72
Longitudinal	1.81 ± 0.98	0.56 (0.34-0.70)	<0.001	0.76 ± 1.20	0.57 (0.35-0.71)	<0.001	0.73 ± 1.04

The measurement for each parameter is shown as mean±SD averaged from all three reads for left ventricular 2D measurements. For inter-reader and intra-reader analysis, the intraclass correlation co-efficient (ICC) along with the 95% confidence intervals (CI) are shown, *p<0.001. The disagreement between reads is reported as the mean±SD absolute difference (|Δ|). Radial measures are obtained from the short-axis view (SAX).

Supplemental Table 4: 3D Left Ventricular Agreement

Factor	Average Measure	Inter-reader			Intra-reader		
		ICC (95% CI)	p-value	Absolute Disagreement |Δ|	ICC (95% CI)	p-value	Absolute Disagreement |Δ|
**Peak Strain (%)**							
Radial	24.7 ± 10.8	0.93 (0.88-0.95)	<0.001	4.0 ± 4.7	0.93 (0.90-0.95)	<0.001	3.6 ± 4.7
Circumferential	-16.7 ± 5.2	0.98 (0.96-0.99)	<0.001	1. 2 ± 1.3	0.98 (0.97-0.99)	<0.001	1.1 ± 1.1
Longitudinal	-10.9 ± 4.0	0.93 (0.89-0.95)	<0.001	1.6 ± 1.6	0.94 (0.91-0.96)	<0.001	1.5 ± 1.4
**Time to Peak Strain (ms)**							
Radial	311± 57	0.80 (0.72-0.86)	<0.001	28 ± 43	0.78 (0.69-0.85)	<0.001	30 ± 45
Circumferential	314 ± 61	0.91 (0.87-0.94)	<0.001	21± 32	0.91 (0.87-0.94)	<0.001	19 ± 32
Longitudinal	333 ± 63	0.72 (0.60-0.80)	<0.001	39 ± 61	0.41 (0.15-0.59)	0.002	47 ± 84
**Systolic Strain Rate (/s)**							
Radial	1.54 ± 0.66	0.82 (0.74-0.88)	<0.001	0.36 ± 0.47	0.76 (0.66-0.83)	<0.001	0.40 ± 0.58
Circumferential	-0.90 ± 0.29	0.94 (0.91-0.96)	<0.001	0.10 ± 0.10	0.95 (0.93-0.97)	<0.001	0.10 ± 0.08
Longitudinal	-0.67 ± 0.21	0.81 (0.73-0.87)	<0.001	0.13 ± 0.14	0.82 (0.74-0.87)	<0.001	0.12 ± 0.12
**Early Diastolic Strain Rate (/s)**							
Radial	-1.73 ± 0.87	0.81 (0.72-0.87)	<0.001	0.44 ± 0.64	0.82 (0.74-0.88)	<0.001	0.45 ± 0.63
Circumferential	0.97 ± 0.38	0.93 (0.90-0.95)	<0.001	0.12 ± 0.16	0.93 (0.90-0.95)	<0.001	0.13 ± 0.16
Longitudinal	0.65 ± 0.26	0.71 (0.57-0.80)	<0.001	0.20 ± 0.21	0.77 (0.67-0.84)	<0.001	0.17 ± 0.19
**Late Diastolic Strain Rate (/s)**							
Radial	-0.54 ± 0.35	0.85 (0.76-0.90)	<0.001	0.18 ± 0.20	0.76 (0.64-0.84)	<0.001	0.19 ± 0.29
Circumferential	0.55 ± 0.27	0.90 (0.85-0.94)	<0.001	0.09 ± 0.14	0.94 (0.91-0.96)	<0.001	0.09 ± 0.10
Longitudinal	0.48 ± 0.20	0.68 (0.50-0.80)	<0.001	0.14 ± 0.20	0.68 (0.51-0.79)	<0.001	0.14 ± 0.15
**Diastolic Strain Rate ratio**							
Radial	4.24 ± 2.51	0.82 (0.70-0.88)	<0.001	1.58 ± 1.53	0.73 (0.59-0.82)	<0.001	1.79 ± 1.98
Circumferential	2.81 ± 1.30	0.86 (0.78-0.91)	<0.001	0.45± 0.77	0.83 (0.75-0.89)	<0.001	0.55 ± 0.86
Longitudinal	1.65 ± 1.03	0.68 (0.50-0.79)	<0.001	0.14 ± 0.57	0.66 (0.49-0.77)	<0.001	0.55 ± 0.68
**Displacement (mm)**							
Radial	4.30 ± 1.35	0.97 (0.96-0.98)	<0.001	0.26 ± 0.36	0.99 (0.98-0.99)	<0.001	0.25 ± 0.36
Longitudinal	5.97 ± 1.98	0.88 (0.83-0.92)	<0.001	0.97 ± 0.96	0.90 (0.86-0.93)	<0.001	0.78 ± 0.74
**Time to Displacement (ms)**							
Radial	309 ± 58	0.82 (0.74-0.88)	<0.001	19 ± 46	0.89 (0.84-0.92)	<0.001	15 ± 36
Longitudinal	327 ± 65	0.70 (0.56-0.79)	<0.001	47 ± 66	0.60 (0.41-0.72)	<0.001	48 ± 74
**Systolic Velocity (m/s)**							
Radial	25.0 ± 8,2	0.93 (0.90-0.95)	<0.001	2.2 ± 4.0	0.97 (0.96-0.98)	<0.001	2.0 ± 1.9
Longitudinal	40.6 ± 136	0.77 (0.67-0.84)	<0.001	9.2 ± 9.4	0.87 (0.82-0.91)	<0.001	6.8 ± 6.7
**Early Diastolic Velocity (m/s)**							
Radial	-26.4 ± 10.0	0.95 (0.92-0.96)	<0.001	2.8 ± 3.7	0.92 (0.88-0.94)	<0.001	3.0 ± 4.9
Longitudinal	-36.5 ± 13.6	0.72 (0.58-0.81)	<0.001	9.6 ± 10.8	0.77 (0.66-0.84)	<0.001	9.8 ± 9.8
**Late Diastolic Velocity (m/s)**							
Radial	-11.7 ± 5.7	0.91 (0.87-0.94)	<0.001	1.9 ± 2.6	0.91 (0.87-0.94)	<0.001	1.7 ± 2.7
Longitudinal	-27.1± 9.6	0.65 (0.45-0.78)	<0.001	7.7 ± 8.3	0.70 (0.55-0.80)	<0.001	6.9 ± 6.5
**Diastolic Velocity ratio**							
Radial	2.92 ± 1.86	0.88 (0.81-0.92)	<0.001	0.60 ± 1.01	0.90 (0.84-0.93)	<0.001	0.57 ± 1.00
Longitudinal	1.54 ± 1.11	0.38 (0.03-0.60)	0.019	0.98 ± 2.52	0.11(-0.34-0.41)	0.284	1.11 ± 2.73

The measurement for each parameter is shown as mean±SD averaged from all three reads for 3D left-ventricular feature tracking. For inter-reader and intra-reader analysis, the intraclass correlation co-efficient (ICC) along with the 95% confidence intervals (CI) are shown, *p<0.001. The disagreement between reads is reported as the mean±SD absolute difference (|Δ|).

Supplemental Table 5: Right Ventricular Agreement

Factor	Average Measure	Inter-reader			Intra-reader		
		ICC (95% CI)	p-value	Absolute Disagreement |Δ|	ICC (95% CI)	p-value	Absolute Disagreement |Δ|
**Peak Strain (%)**							
Radial (LAX)	40.1 ± 16.3	0.80 (0.70-0.87)	<0.001	8.7 ± 10.3	0.87 (0.80-0.91)	<0.001	7.6 ± 9.4
Longitudinal	-18.5 ± 5.4	0.84 (0.75-0.89)	<0.001	2.7 ± 3.0	0.91 (0.87-0.94)	<0.001	2.1 ± 2.4
**Time to Peak Strain (ms)**							
Radial (LAX)	310 ± 64	0.62 (0.43-0.75)	<0.001	36 ± 58	0.68 (0.53-0.78)	<0.001	42 ± 72
Longitudinal	303 ± 58	0.66 (0.49-0.77)	<0.001	33 ± 49	0.71 (0.58-0.81)	<0.001	34 ± 64
**Systolic Strain Rate (/s)**							
Radial (LAX)	2.36 ± 1.02	0.68 (0.53-0.79)	<0.001	0.64 ± 0.77	0.81 (0.71-0.87)	<0.001	0.65 ± 0.63
Longitudinal	-1.21 ± 0.45	0.69 (0.53-0.79)	<0.001	0.30 ± 0.44	0.87 (0.81-0.91)	<0.001	0.27 ± 0.28
**Early Diastolic Strain Rate (/s)**							
Radial (LAX)	-2.52 ± 1.23	0.81 (0.71-0.87)	<0.001	0.74 ± 0.78	0.86 (0.79-0.91)	<0.001	0.65 ± 0.64
Longitudinal	0.99 ± 0.38	0.86 (0.80-0.91)	<0.001	0.21 ± 0.20	0.85 (0.77-0.90)	<0.001	0.20 ± 0.20
**Late Diastolic Strain Rate (/s)**							
Radial (LAX)	-1.29 ± 0.75	0.91 (0.85-0.95)	<0.001	0.34 ± 0.35	0.82 (0.72-0.89)	<0.001	0.46 ± 0.46
Longitudinal	0.88 ± 0.45	0.86 (0.77-0.92)	<0.001	0.25 ± 0.30	0.88 (0.81-0.93)	<0.001	0.24 ± 0.20
**Diastolic Strain Rate ratio**							
Radial (LAX)	2.59 ± 1.83	0.74 (0.55-0.85)	<0.001	0.77 ± 1.27	0.79 (0.66-0.87)	<0.001	0.84 ± 1.02
Longitudinal	1.39 ± 0.90	0.69 (0.47-0.81)	<0.001	0.54 ± 0.87	0.78 (0.64-0.86)	<0.001	0.50 ± 0.73
**Displacement (mm)**							
Radial (LAX)	5.35 ± 2.20	0.91 (0.87-0.94)	<0.001	0.86 ± 0.98	0.94 (0.91-0.96)	<0.001	0.65 ± 0.87
Longitudinal	6.63 ± 2.88	0.92 (0.87-0.94)	<0.001	1.29 ± 1.19	0.94 (0.90-0.96)	<0.001	1.00 ± 1.06
**Time to Displacement (ms)**							
Radial (LAX)	291 ± 47	0.69 (0.54-0.79)	<0.001	18 ± 48	0.82 (0.74-0.88)	<0.001	19 ± 37
Longitudinal	318 ± 59	0.61 (0.43-0.74)	<0.001	34 ± 59	0.81 (0.72-0.87)	<0.001	28 ± 50
**Systolic Velocity (m/s)**							
Radial (LAX)	33.2 ± 14.2	0.92 (0.88-0.95)	<0.001	5.6 ± 5.9	0.92 (0.89-0.95)	<0.001	5.0 ± 5.5
Longitudinal	45.1 ± 17.4	0.83 (0.74-0.88)	<0.001	10.0 ± 10.8	0.89 (0.84-0.93)	<0.001	8.0 ± 8.4
**Early Diastolic Velocity (m/s)**							
Radial (LAX)	-31.7 ± 13.4	0.91 (0.87-0.94)	<0.001	5.8 ± 6.0	0.92 (0.88-0.95)	<0.001	4.9 ± 5.4
Longitudinal	-35.1 ± 16.4	0.88 (0.81-0.92)	<0.001	7.3 ± 7.6	0.90 (0.84-0.93)	<0.001	6.5 ± 7.4
**Late Diastolic Velocity (m/s)**							
Radial (LAX)	-13.5 ± 8.8	0.88 (0.77-0.93)	<0.001	4.2 ± 4.5	0.93 (0.88-0.96)	<0.001	3.4 ± 3.3
Longitudinal	-32.6 ± 16.9	0.93 (0.88-0.96)	<0.001	7.4 ± 5.0	0.95 (0.92-0.97)	<0.001	5.5 ± 5.3
**Diastolic Velocity ratio**							
Radial (LAX)	3.55 ± 4.90	0.71 (0.46-0.85)	<0.001	0.84 ± 1.36	0.90 (0.82-0.94)	<0.001	0.69 ± 0.61
Longitudinal	1.35 ± 1.03	0.78 (0.63-0.87)	<0.001	0.40 ± 0.44	0.89 (0.82-0.93)	<0.001	0.28 ± 0.36

The measurement for each parameter is shown as mean±SD averaged from all three reads for right ventricular feature tracking. For inter-reader and intra-reader analysis, the intraclass correlation co-efficient (ICC) along with the 95% confidence intervals (CI) are shown, *p<0.001. The disagreement between reads is reported as the mean±SD absolute difference (|Δ|). Radial measures are obtained from the long-axis view (LAX).
